# Factors Affecting Element Concentrations in Eggshells of Three Sympatrically Nesting Waterbirds in Northern Poland

**DOI:** 10.1007/s00244-017-0481-y

**Published:** 2017-11-23

**Authors:** Ignacy Kitowski, Dariusz Jakubas, Piotr Indykiewicz, Dariusz Wiącek

**Affiliations:** 1 State School of Higher Education in Chełm, Pocztowa 54, 22-100 Chełm, Poland; 2 Department of Vertebrate Ecology and Zoology, Faculty of Biology, University of Gdańsk, Wita Stwosza 59, 80-308 Gdańsk, Poland; 3 Department of Biology and Animal Environment, University of Sciences and Technology, Kordeckiego 20, 85-225 Bydgoszcz, Poland; 4 Institute of Agrophysics, Polish Academy of Sciences, Doświadczalna 4, 20-290 Lublin, Poland

## Abstract

Avian eggshells are convenient samples in biomonitoring studies, because they are easily accessible, especially from colonially or semicolonially breeding birds. In the present study, concentrations of 17 elements, including heavy metals and essential elements in post-hatch eggshells, were compared among three species of waterbirds of differing strategies for gaining reserves for egg production and diet: mallard, *Anas platyrhynchos* (ML, a capital breeder, mainly herbivorous), common tern, *Sterna hirundo* (CT, an income breeder, piscivorous) and black-headed gull, *Chroicocephalus ridibundus* (BHG, mixed strategy, omnivorous) and breeding sympatrically in three sites in North Poland. Analyses revealed that Fe, Zn, and Cu levels differed the most in the studied species, which may be explained by various contributions of fish, aquatic plants, and soil invertebrates in their diets. Generally, the studied species’ eggshells accumulated amounts of elements comparable to those reported for other waterbirds without putting the growth and development of the embryo at risk. The only exception was very high levels of Cr in ML and CT, which may be explained by their foraging on aquatic organisms in waterbodies polluted by this element. Intersite differences in eggshell concentrations of Ni, Sr, Hg and Cr in CT (an income breeder) may be explained by the influence of local pollution sources (small factories, polluted river).

**Electronic supplementary material:**

The online version of this article (10.1007/s00244-017-0481-y) contains supplementary material, which is available to authorized users.

Avian eggshells are commonly used in studies focusing on bioindication and environmental monitoring (Lam et al. 2005; Ayas et al. 2008; Kim and Oh 2014; Khademi et al. 2015; Simonetti et al. 2015). Post-hatch eggshells are useful samples in biomonitoring studies, because they are easily accessible, especially from colonially or semicolonially breeding birds (Fu et al. 2014). Eggs are formed during a restricted period by adult females, which reduces the sources of variability (Sánchez-Virosta et al. 2015). During egg formation, females remove some contaminants from their bodies by sequestering them in the eggshells (Burger and Gochfeld 1996; Migula et al. 2000; Orłowski et al. 2014; Luo et al. 2016). However, the relationships among the various elements in the shells and contents vary between embryonated and non-embryonated eggs (Orłowski et al. 2016). The greater number of significant correlations has been found for embryonated eggs, which may be explained by the mobilization of elements (primarily accompanying intensive Ca resorption) from the shells to the egg contents (Orłowski et al. 2017). The eggshell serves as the major source of both Ca and Mg for the developing embryo (Packard and Packard 1991). Thus, ignoring the shell as a source of elements or in the element budget of the embryo undoubtedly prevents proper conclusions about the flux and bioaccumulation of contaminants in avian embryos (Orłowski et al. 2016).

Signals from eggshells reflect a short period of time (prelaying) and various spatial scales depending on the strategy for gaining nutrients for the eggs’ production: capital breeders store nutrients before breeding, while income breeders obtain nutrients daily during the prelaying period (Stephens et al. 2009). Thus, the income breeders’ levels of trace elements in eggs reflect contamination in the local breeding grounds, but in capital breeders, trace elements reflect contamination in wintering areas or stopover sites during spring migration.

This study focused on the concentrations of heavy metals and other elements in post-hatch eggshells of three sympatrically nesting waterbirds: black-headed gull, *Chroicocephalus ridibundus* (BHG), common tern, *Sterna hirundo* (CT), and mallard *Anas platyrhynchos* (ML), which differ in their strategies for gaining reserves for egg production and diet composition.

Mallard *Anas platyrhynchos* is an omnivorous duck. It is mainly herbivorous and granivorous, but it supplements its diet with locally available food, including aquatic invertebrates, fish, and amphibians, and in urban areas food waste of anthropogenic origin, such as bread (Cramp and Simmons 1977; Green and Selva 2000; Soons et al. 2016). Regarding its egg-formation strategy, ML is classified as a capital breeder (Boos et al. 2002; Butler and McGraw 2013).

The common tern, *Sterna hirundo*, is an opportunistic predator that changes prey as well as foraging behavior depending on local conditions (Bukacinski and Bukacinska 2015). It preys on fish and aquatic invertebrates, mainly crustaceans and insects (Becker and Ludwigs 2011; Bukacinski and Bukacinska 2015). Among the studied avian species, fish is the CT’s most important diet component (Grajewska et al. 2015; Bukacinski and Bukacinska 2015; Indykiewicz P, personal communication). Regarding its egg-formation strategy, CT is classified as an income breeder (Bond and Diamond 2010), obtaining nutrients on the breeding grounds during the prelaying period.

The black-headed gull, *Chroicocephalus ridibundus*, is an omnivorous gull with a diet consisting of invertebrates, especially earthworms, plants, and fish, and in urban areas, anthropogenic food waste (Vernon 1972; Cuendet 1983; Kitowski et al. 2017; Indykiewicz P., unpublished data). Regarding egg-formation, BHG adopts a mixed strategy to gain nutrients for egg production. Some nutrients are endogenous reserves acquired before breeding, whereas others are acquired near the breeding colony (Klaassen et al. 2004; Stephens et al. 2009).

The purpose of this study was to compare concentrations of heavy metals and other elements in post-hatch eggshells between species and sites. Considering the interspecies differences in diet composition and strategies for gaining nutrients for egg formation (Boos et al. 2002; Bond and Diamond 2010; Butler and McGraw 2013), interspecies differences in accumulating elements in eggshells were expected. Contamination of CT, ML, and BHG eggshells should indicate contamination in areas close to the breeding site, the last stopover site, or a mix of both areas, respectively. Given the common phenomenon of unintentionally swallowing lead shot pellets (mistaken for grit) by ML (Pain 1990; Szymczyk and Zalewski 2003; Mateo 2009), higher Pb concentrations are expected in the eggshells of this species compared with BHG and CT. Considering the high prevalence of fish in the diet of CT, higher concentrations of Cu and Zn may be expected compared to the more herbivorous ML. Considering the intersite differences in land cover, high levels of Cu and Zn in areas with high contributions from water bodies may be expected. Fish serves as the important source of those elements for piscivorous vertebrates (Radwan et al. 1990; Łuczyńska et al. 2009). Given that a diet rich in fish favors Hg accumulation in eggs, including the eggshells (Monteiro and Furness 1995; Grajewska et al. 2015; Ackerman et al. 2016), piscivorous CT and partly piscivorous BHG are predicted to have higher Hg levels in their eggshells. Given the intersite differences in habitat composition among the studied breeding sites (Table ES1), intersite differences in the element concentration in eggshells of the CT, the only species gaining reserves for egg production locally, may be expected. Moreover, high Cu concentrations (due to high concentrations in aquatic prey) in eggshells in sites with extensive water bodies (Skoki Duże) and a high proportion of elements originating from fertilizers (e.g., Cd) in sites with extensive farmlands (Pakość) may be expected.

## Materials and Methods

### Study Area

Post-hatch eggshells were collected in spring 2015 in three sites in North (N) Poland, at Skoki Duże, Pakość, and Koronowo, where all studied species nest sympatrically (Table 1).

**Table 1:** Characteristics of the colonies of three sympatric species: black-headed gull (BHG), mallard (ML), and common tern (CT)

Colony location	Estimated no. of breeding pairs	Colony characteristic	Local sources of contamination
Koronowo 53°20.069′ 17°57.884′	BHG 130–150 CT 20–25 ML 13–17	Studied species breed on a small islet in the northern part of Koronowskie Lake, an artificial mesotrophic reservoir established late in the 1960s. The lake is used for recreation, and seasonal holiday houses are located near the island	The lake is used for recreation, and seasonal holiday houses are located near the island. Local sources of contamination are pesticides and the effects of use of the nearby gardening allotments and municipal sewage from holiday houses and the town of Koronowo town (12,000 inhabitants)
Pakość 52°46.973′ 18°05.056′	BHG 700–1200 CT 35 ML 25–30	Situated on the steep banks of islets in the mesotrophic Pakoskie Północne Lake; each island supports a metal pillar for a cable car system; the lake is surrounded by vast arable fields	Local sources of contamination include the intensive use of manure and agrochemicals which are regularly used in farmland around the lake. Residues of these contaminants flow from arable land to the lake basin. Close location of tannery and small factory processing steal and alloys for industry and mining
Skoki Duże 52°36.399′ 19°23.643′	BHG 800–1300 CT 197–330 ML 45–55	The studied species breed on two sandy islets overgrown with grass in a deep oligotrophic artificial waterbody in a functioning gravel pit. The site is surrounded by agricultural areas and a small, deciduous woodland. The site is protected as Area in the Natura 2000 network (PLB040005)	Fumes emitted from machinery and pollution from conveyor belts and the intensive traffic of heavy dump trucks. The nearest factories: a large petrochemical operation in Płock (18 km) and a chemical plant (nitrogen fertilizers and PVC in Włocławek (31 km)

**Fig. 1:**
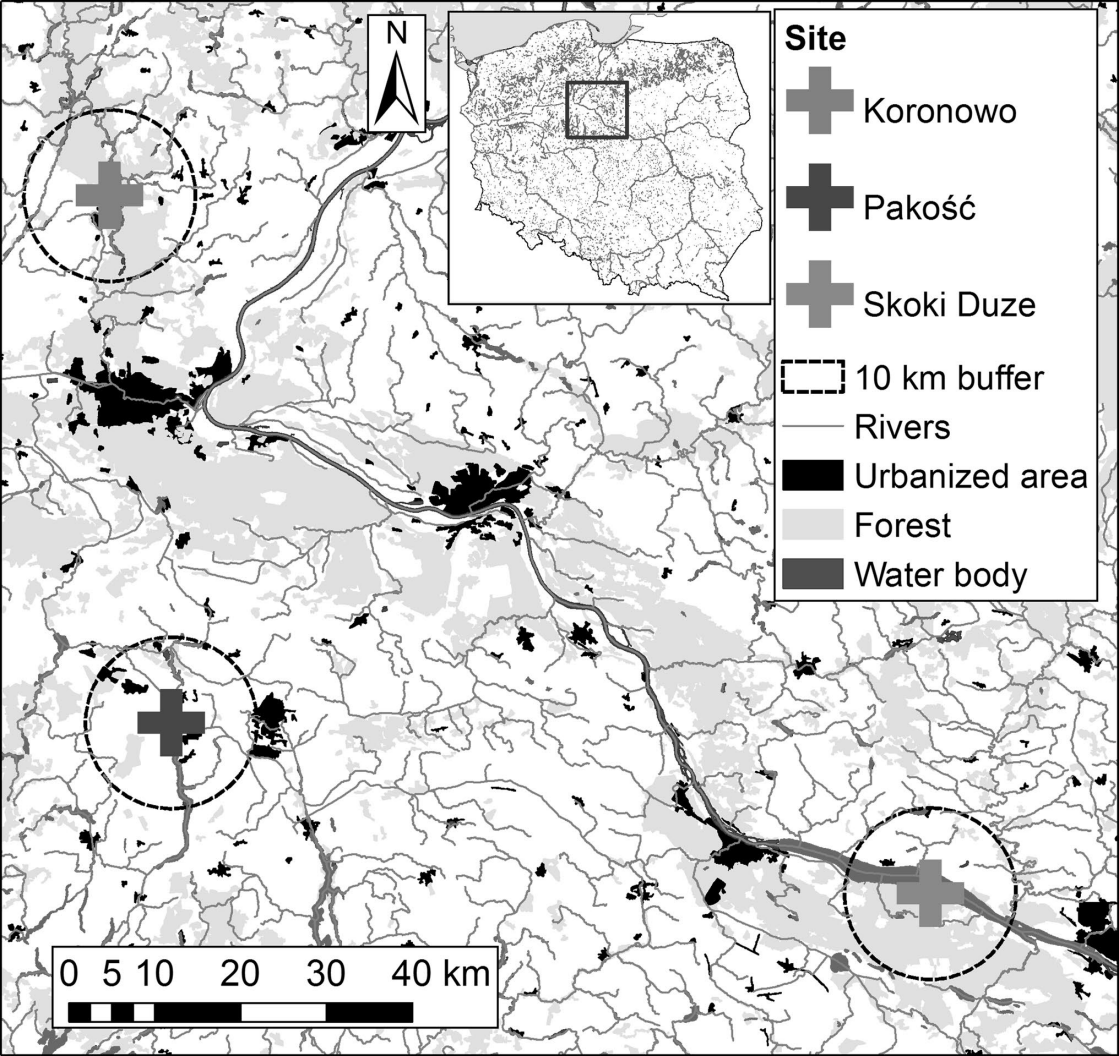
Study area with the location of all studied sites (crosses) with 10-km buffers (dashed line), selected land cover types [according to the Corine Land Cover (CLC2006) model (http://www.eea.europa.eu/, EEA Copenhagen, 2012)], and the nearest factories (pollution emission sources)

Most of the studied sites are located in areas with a prevalence of agricultural land (Fig. 1; Table ES1). Such a land structure was most marked at Pakość, where agricultural land constituted 85.8% of the area within 10 km (Fig. 1; Table ES1). Negligible aquatic habitats covered the 10-km zones around the sites (Table ES1), except for Skoki Duże, where the Włocławek Reservoir (an artificial water body established after building the dam on the Vistula River) constituted 11.2% of the surrounding 10-km zone.

Throughout the breeding season BHG feed mostly around the water bodies where their colonies are located, except at Pakość, where they feed mostly in arable fields. At Skoki Duże, the gulls forage in the Vistula River valley. Rubbish dumps are located within approximately 2 km of each colony that we monitored, except for the Skoki Duże colony. These dumps serve as an additional source of food for BHG (Indykiewicz P., unpublished data). ML and CT generally forage in the water bodies where they breed. CT sometimes forage in other nearby aquatic habitats (e.g., in the Vistula River at Skoki Duże). ML also forage in farmlands near the breeding site.

### Field Methods

The black-headed gull laid eggs between the first week of April and first week of May, ML between the first half of April and the first half of June, and CT from the beginning to the end of May. BHG and CT nests were monitored every 2–3 days, ML every 3–4 days, and the post-hatched eggshells were collected. Eggshells were searched for within 3–4 m of the nest as soon as the chicks hatched. The collected eggshells were preliminarily cleaned of larger dirt, such as soil and feces, and then were placed in airtight containers and delivered to the laboratory. In total, 35 post-hatch BHG eggshells were collected (12 at Skoki Duże, 12 at Pakość, and 11 at Koronowo), 34 ML (11 at Skoki Duże, 11 at Pakość, and 12 at Koronowo), and 36 CT (12 at Skoki Duże, 12 at Pakość, and 12 at Koronowo).

### Analytical Procedure

Upon delivery to the laboratory the inner membrane of the eggshells was removed. Eggshells were washed with deionized water, rinsed with acetone, and ground in a ceramic mortar before measurements. All glassware and utensils was soaked in an acid bath (5 M HNO_3_) for 24 h, rinsed with demineralized water, and dried under a laminar flow hood before use to minimize the risk of metal contamination. Samples (500 ± 1 mg) were mixed with 10 mL of concentrated HNO_3_ (Sigma Aldrich, Chempur, Poland) and wet ashed. Mineralization was carried out in a Microwave Digestion System with optical, temperature, and pressure monitoring of each sample during acid digestion (Berghof Speedwave, Eningen, Germany) in Teflon vials (type DAP 100). For mineralization details, see electronic supplementary material (ES2). The clear elemental solution obtained after mineralization was cooled to room temperature, transferred to 50-mL flasks, and filled with demineralized water (ELGA Pure Lab Classic) to the indicated level. An iCAP Series 6500 inductively coupled plasma optical emission spectrometer (Thermo Scientific, USA) equipped with a charge injection device (CID) was used for element detection (iCAP 2010). The spectrometer was controlled with PC-based iTEVA software (see instrumental parameters in ES2).

Considering the mineralization method (dilution of 500 mg of sample in 10 mL of HNO_3_ with a density of 1.51 g cm^−3^), the limit of Hg detection was estimated to 0.058 µg L^−1^ (3.72 × 10^−5^ mg kg^−1^).

Samples were run in batches (colonies) and each colony included a blank (control) sample. A certified reference material, TraceCERT – Periodic table mix 1 for ICP (Fluka Analytical, Sigma Aldrich), was used to control the accuracy of the method under existing working conditions. Validation of the analytical method is described in ES2. All concentrations obtained in this study are given in mg kg^−1^ dry weight (dw).

### Statistical Analyses

To investigate variations in the qualitative and quantitative composition of trace elements in post-hatch eggshells, we used principal component analysis (PCA). This technique was applied to reduce the number of variables to a few new factors representing groups of elements with significantly correlated concentrations.

To compare the qualitative and quantitative compositions of all trace elements in eggshells among the studied species and sites, we applied following multivariate methods:

(1) Multivariate (for all elements together) PERMANOVA (nonparametric MANOVA based on the Bray–Curtis measure; Anderson 2001) with fixed factors (age and sex) and their interaction as explanatory variables; when the interaction effect was significant, intersite differences were compared exclusively for the income breeder, CT; (2) The similarity percentage breakdown (SIMPER) procedure to assess the average percentage contribution of individual factors to the dissimilarity between objects in a Bray–Curtis dissimilarity matrix (Clarke 1993); (3) Univariate analysis (for particular elements) using one-way PERMANOVA (nonparametric MANOVA based on the Bray–Curtis measure; Anderson 2001) with fixed factors (colony and sex) and their interaction as explanatory variables.

We assessed whether the data sufficiently met the assumptions of the linear model using Q–Q plots (quantile expected in normal distribution vs quantile observed plot for residuals). As the distribution of the obtained data was not normal, a log(*x* + 1) transformation was used resulting in residuals with a normal distribution. Statistical analyses were conducted using STATISTICA 12.0 (StatSoft, Inc. 2014) and PAST 3.0 (Hammer et al. 2001).

## Results

Variations in the qualitative and quantitative composition of trace elements in post-hatch eggshells

**Fig. 2:**
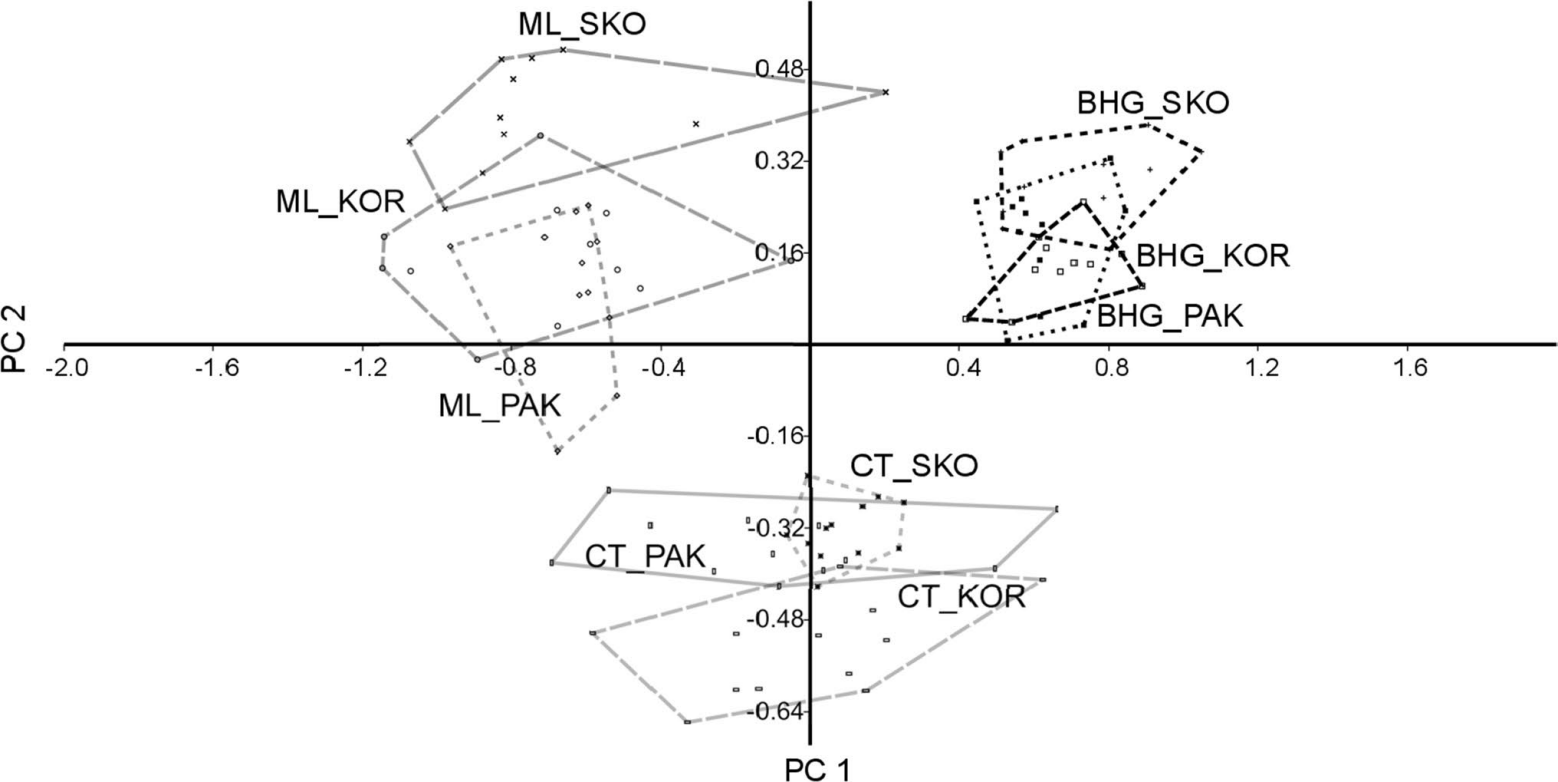
PCA plot showing elemental concentrations in the eggshells of black-headed gulls (BHG), mallards (ML), and common tern (CT) breeding in Koronowo (KOR), Pakość (PAK), and Skoki Duże (SKO). Convex hulls contain all samples from one species and breeding site

Principal component analysis (PCA) revealed that 82.3% of the total variance was explained by the three axes (Table 2). PC1 explained 53.6% of the total variance and was moderately positively correlated with Fe (*r* = 0.66; Table 2). PC2 explained 14.9% of the total variance and was highly positively correlated with Sr (*r* = 0.67). PC3 explained 13.7% of the total variance and was moderately positively correlated with Fe (*r* = 0.67) and Mn (*r* = 0.55; Table 2). All of the studied species clustered in various positions in the PCA plot (Fig. 2). All of the BHG samples were the most clustered (Fig. 2).

**Table 2:** Values of principal component loadings for the studied elements in the eggshells of the examined species; moderately correlated values (*r* > 0.05) bolded

Elements	PC 1	PC 2	PC 3
As	0.09	0.11	− 0.04
Ca	0.25	0.23	− 0.19
Cd	0.00	0.00	0.01
Cr	− 0.22	− 0.41	0.18
Cu	− 0.36	− 0.17	0.27
Fe	**0.66**	− 0.10	**0.67**
Hg	− 0.05	− 0.01	0.04
Mg	0.17	− 0.19	− 0.13
Mn	− 0.18	0.39	**0.55**
Mo	0.01	0.01	− 0.01
Ni	− 0.05	0.15	0.05
Pb	− 0.06	0.06	0.07
Sc	− 0.03	0.03	0.03
Se	0.13	− 0.07	− 0.07
Sr	− 0.03	**0.67**	− 0.04
V	− 0.18	0.21	0.12
Zn	0.43	0.05	− 0.23
Eigen values	0.36	0.10	0.09
Total variance explained (%)	53.6	14.9	13.7

### Intergroup Differences: All Elements Combined

The concentrations of all combined studied elements were significantly affected by species (multivariate two-way PERMANOVA, similarity measure: Bray–Curtis, *F*_2,104_ = 146.1, *p* = 0.0001), breeding site (*F*_2,104_ = 8.40, *p* = 0.0001), and species × site interaction (*F*_2,104_ = − 2.55, *p* = 0.0001). SIMPER analysis showed that Fe, Zn, and Cu contributed the most (17, 12, and 10%, respectively) to the pattern of overall dissimilarity observed in elemental concentrations (Table 3). Regarding the species × site interaction effect, one-way PERMANOVA indicated that elemental concentrations did not differ significantly among the BHG breeding sites (*p* > 0.11) or between the CT colonies at Pakość and Skoki Duże (*p* = 0.23). All of the other differences were significant (Table 4). SIMPER analysis showed that the following elements contributed the most (> 10%) to the pattern of interspecies dissimilarity: Fe, Zn, and Cu for ML-BHG; Fe, Cr, Cu, Ca, Sr, and Zn for BHG-CT; and Fe, Mn, Sr, and Zn for ML-CT (Table 3).

**Table 3:** Sources of variability in the elemental concentrations [log(*x* + 1) transformed] (average percentage dissimilarity) in the eggshells of three species of waterbirds from the studied sites according to a SIMPER analysis; only elements with a contribution > 10% are shown

Element	Average dissimilarity	Contribution (%)
*Overall dissimilarity*		
Fe	1.715	17.1
Zn	1.224	12.2
Cu	1.027	10.3
*Interspecies*—*BHG*-*ML*		
Fe	2.5	17.2
Zn	2.0	13.6
Cu	1.7	12.1
*Interspecies*—*BHG*-*CT*		
Fe	1.4	14.5
Cr	1.4	13.8
Cu	1.3	12.8
Ca	1.3	12.7
Sr	1.2	11.8
Zn	1.1	11.5
*Interspecies*—*ML*-*CT*		
Fe	1.9	18.0
Mn	1.5	14.4
Sr	1.3	12.6
Zn	1.1	10.5

Species codes: *BHG* black-headed gull, *ML* mallard, *CT* common tern

**Table 4:** Intergroup differences (one-way PERMANOVA, *p* values) in elemental concentration in eggshells of black-headed gulls (BHG), mallards (ML) and common tern (CT) breeding at Koronowo (KOR), Pakość (PAK), and Skoki Duże (SKO)

Species/sites	BHG SKO	BHG KOR	BHG PAK	ML SKO	ML KOR	ML PAK	CT SKO	CT KOR	CT PAK
BHG_SKO		0.212	0.295	**0.004**	**0.004**	**0.004**	**0.004**	**0.004**	**0.004**
BHG_KOR	0.212		0.112	**0.004**	**0.004**	**0.004**	**0.004**	**0.004**	**0.004**
BHG_PAK	0.295	0.112		**0.004**	**0.004**	**0.004**	**0.004**	**0.004**	**0.004**
ML_SKO	**0.004**	**0.004**	**0.004**		**0.004**	**0.004**	**0.004**	**0.004**	**0.004**
ML_KOR	**0.004**	**0.004**	**0.004**	**0.004**		**0.004**	**0.004**	**0.004**	**0.004**
ML_PAK	**0.004**	**0.004**	**0.004**	**0.004**	**0.004**		**0.004**	**0.004**	**0.004**
CT_SKO	**0.004**	**0.004**	**0.004**	**0.004**	**0.004**	**0.004**		**0.004**	0.227
CT_KOR	**0.004**	**0.004**	**0.004**	**0.004**	**0.004**	**0.004**	**0.004**		**0.036**
CT_PAK	**0.004**	**0.004**	**0.004**	**0.004**	**0.004**	**0.004**	0.227	**0.036**	

Significant (*p* < 0.05) values bolded

### Intergroup Differences: Particular Elements

Univariate PERMANOVA analyses performed separately for particular elements revealed that the levels of As, Cd, Cu, Fe, Mo, and Pb in post-hatch eggshells were significantly affected only by species. Other studied factors were insignificant (Table ES3). The Mn and V levels in post-hatched eggshells were significantly affected by both species and site factors (Table ES3).

The As concentrations differed significantly among all species (*p* = 0.003) with the highest value in ML and the lowest in CT. Significantly higher levels of Cd were found in ML compared with CT (*p* = 0.0003) and BHG (*p* = 0.03; Table ES4). All of the species differed significantly with respect to Cu (*p* = 0.003), with the highest value in ML and the lowest in BHG. All of the species differed significantly (*p* < 0.01) in Fe, with the highest value in BHG and the lowest in ML (Table ES4). Levels of Mo in all species differed significantly (*p* < 0.004), with the highest value in BHG and the lowest in CT (Table ES4). Concentrations of Pb differed significantly between ML and BHG (*p* = 0.0003) and ML and CT, with the highest values in ML. The levels of Pb in CT and BHG were similar (*p* = 1.0; Table ES4).

Mn levels differed significantly among the studied species (*p* < 0.004), with the highest value in ML and the lowest in CT (Table ES4). Regarding the site effect, Mn concentrations at Pakość tended to be lower than at Koronowo (*p* = 0.055; Table ES5). Concentrations of V differed significantly among all studied species (*p* = 0.003), with the highest value in ML and the lowest in CT (Table ES4). The site effect was not significant (all *p* > 0.17).

One-way PERMANOVA revealed that the concentrations of the remaining elements (Ca, Cr, Hg, Mg, Ni, Sc, Se, Sr, and Zn) in post-hatch eggshells were significantly affected by interactions between species and site (species × site). It was accompanied by a significant effect of species (Mg, Zn) or both species and site (Ca, Cr, Hg, Ni, Sc, Se, Sr; Table ES3).

To test the formulated hypotheses, further analyses of these elements were focused on interspecies differences and intersite differences exclusively for the income breeder, CT.

Regarding interspecies differences, Mg concentrations differed significantly between ML and BHG (*p* = 0.0003) and ML and CT (*p* = 0.0003), with the lowest level in ML. Concentrations in CT and BHG were similar (*p* = 1.0; Table ES4). Levels of Ca in all species differed significantly among the species (*p* = 0.0003), with the highest value in BHG and the lowest in ML (Table ES4). Zn concentration differed significantly among all species (*p* = 0.0003), with the highest value in BHG and the lowest in ML (Table ES4). Cr concentrations differed significantly among all species (*p* < 0.002), with the highest value in CT and the lowest in BHG (Table ES4). Hg levels differed significantly among all species studied (*p* = 0.0003), with the highest value in ML and undetectable values in BHG (Table ES4). Ni concentrations differed significantly among all species (*p* = 0.0003), with the highest value in ML and lowest in CT (Table ES3). Sc concentrations in CT were considerably higher than in BHG (*p* = 0.0003) and ML (*p* = 0.001; Table ES4). Se concentration differed significantly among all species (*p* = 0.0003), with the highest value in BHG and the lowest in ML (Table ES3). Sr levels in CT were considerably lower than in BHG (*p* = 0.0003) and ML (*p* = 0.0003; Table ES4).

### Intersite Differences in the Income Breeder, CT

Univariate PERMANOVA revealed lack of significant interaction effect for As, Cd, Cu, Fe, Mn, Pb, and V (Table ES3). For elements with significant species × site interaction effect (*p* > 0.05), there were no significant intersite differences for CT in the concentrations of Ca (*p* > 0.65), Mg (*p* = 1.0), Sc (*p* > 0.12), or Zn (*p* = 1.0). The Cr values at Koronowo were significantly lower than at Pakość (*p* = 0.04; Table ES5). The same pattern was found for Hg, with significantly lower values in CT eggshells from Koronowo compared with Pakość (*p* = 0.01; Table ES5). Eggshell Ni concentrations at Koronowo were significantly lower than at Pakość (*p* = 0.004) and Skoki Duże (*p* = 0.004; Table ES5). The Se values at Skoki Duże were significantly higher compared to Pakość (*p* = 0.004) and Koronowo (*p* = 0.007; Table ES5). Significantly lower Sr concentrations in CT were found in Koronowo compared with Pakość (*p* = 0.003) and Skoki Duże (*p* = 0.004; Table ES5).

## Discussion

To our knowledge, this is the first study to compare elemental concentrations in post-hatch eggshells collected in one area from three species of sympatrically breeding waterbirds adopting various strategies of obtaining nutrients for egg formation.

Interspecies differences in concentrations of selected essential elements (iron, zinc, copper, calcium, manganese)

Fe and Zn contributed considerably (> 10%) to all pairs of interspecies dissimilarities (Table 3). Fish tissues are an important source of Zn and Fe (Radwan et al. 1990; Łuczyńska et al. 2009), which may explain the high values of these elements in partly piscivorous BHG and their low values in mainly herbivorous/granivorous ML (Table ES4). Conversely, one should expect the highest Fe and Zn values in piscivorous CT. However, CT is an opportunistic predator that may switch from fish to other prey (Becker and Ludwigs 2011; Bukacinski and Bukacinska 2015). Thus, we cannot exclude that the relatively late arrival of CT to the breeding colony before egg formation may supplement their piscivorous diet with invertebrates, which results in lower levels of those elements in eggshells than expected. Zn concentration was the highest in BHG, which frequently forage on earthworms (Cuendet 1983), known for their high absorption of this essential element [concentrations (mg kg^−1^ dw) for species occurring in Poland: *Lumbricus terrestris*—790–1066, *Aporrectodea caliginosa*—401–1530, and *Eisenia fetida*—450–617 (Łaszczyca et al. 2004)]. Natural and mineral fertilizers serve as an important source of the total annual input of Zn into agricultural soils (Nicholson et al. 2003). Similar high Zn concentrations in eggshells of the same species have been reported for other parts of Poland (Table ES6, Table ES7).

Cu contributed considerably to the dissimilarity for the species pairs BHG-ML and BHG-CT (Table 3). Aquatic plants and fish serve as the most important sources of this element. High Cu accumulation in internal organs has been reported for piscivorous avian species (Nam et al. 2005; Horai et al. 2007; Skoric et al. 2012) and herbivorous anatids (Schummer et al. 2011; Komosa et al. 2012). The eggshell Cu concentration pattern (ML > CT > HG) probably reflects different contributions of those food types in the diets of the studied species, with a high contribution of aquatic plants in herbivorous/granivorous ML, a high contribution of fish in CT, and the lowest contribution of aquatic food in BHG.

Ca—the most important element to affect eggshell structure—contributed considerably to the interspecies dissimilarity in elemental concentration in the eggshells for the species pair BHG-CT (Table 3), with the highest concentration in the former species (Table ES4). Those high values in BHG may have resulted from a high-diversity diet consisting of important Ca sources, such as fish (Łuczyńska et al. 2009; Lidwin-Kazmierkiewicz et al. 2009), plants (Schierup and Larsen 1981; Brix and Lyngby 1983; Obolewski et al. 2010), or invertebrates (earthworms and mollusks) (Wheeler 1992; Morgan and Morgan 1991, 1999; Jurkiewicz-Karnkowska 2005). The low concentrations of Ca in CT (Table ES4) may be attributed to less frequent grit ingestion and a less diverse diet consisting mainly of fish compared with BHG in studied colonies (Indykiewicz P, unpublished data).

Similar to Zn, proper amounts of Mn have a positive impact on eggshell density, thickness, and the hatchability of eggs (Leach and Gross 1983; Swiatkiewicz and Koreleski 2008). The Mn concentration in the post-hatch eggshells of the studied ML (3.61 mg kg^−1^) was the highest reported for waterbirds (Table ES6, Table ES7). As in the case of Cu, the observed Mn accumulation sequence ML > BHG > CT (Table ES4) reflects the contribution of aquatic plants in the birds’ diet. This kind of food is rich in Mn (Samecka-Cymerman and Kempers 2000; Demirezen and Aksoy 2006; Parzych et al. 2016) as water plants are able to accumulate up to 500 mg kg^−1^ of Mn without negative effects (Allen 1989).

### Interspecies Differences in Concentrations of Selected Heavy Metals and Strontium

Sr contributed considerably to inter-species dissimilarity in elemental concentrations in the eggshells for the species pairs BHG-CT and ML-CT (Table 3). The very low concentrations of this element observed in CT eggshells (Table ES4) may be attributed to less frequent grit (gastrolith) ingestion in this mainly piscivorous tern. Grit serves as an important source of Ca during egg formation (Bendell-Young and Bendell 1999; Gionfriddo and Best 1999; Sherfy et al. 2001). Sr is strongly associated with Ca metabolism; thus, higher Ca requirements for the female during egg production result in increased Ca absorption as well as increased absorption of Sr (Kottferova et al. 2001; Mora 2003).

In agreement with expectations, ML eggshells accumulated 32 times more Pb than the other studied species (Table ES4). However, all of those values were relatively low compared with those reported for other waterbird species (up to 88.5 mg kg^−1^ dw; Table ES6, Table ES7). Lead shot pellets are commonly swallowed as grit by mistake, which is the common source of Pb contamination in ducks, including MLs (Figuerola et al. 2005; Martinez-Haro et al. 2011). Fertilizer runoff from the soil to water bodies may serve as an additional source of Pb contamination. Fertilizers often are contaminated by this element (Aro et al. 1998; McBride and Spiers 2001; Nziguheba and Smolders 2008; Bodnar et al. 2016).

In contrast to our expectations regarding high levels of Hg in piscivorous CT and partly piscivorous BHG, detectable concentrations of this element were found only in the eggshells of CT and ML (Table ES4). The lack of detectable concentrations of Hg in the eggshells of BHG may be explained by the frequent foraging of BHGs on anthropogenic food in winter that favor lower accumulations of Hg (Kitowski et al. 2015; Peterson et al. 2017) compared with foraging on fish favoring Hg accumulation in eggs, including eggshells (Monteiro and Furness 1995; Grajewska et al. 2015; Ackerman et al. 2016). Conversely, birds may have foraged in areas contaminated with Hg, but this element may have been allocated to the egg content as has been reported previously for the black-tailed gull, *Larus crassirostris* (Agusa et al. 2005).

### Intersite Differences in Elemental Concentrations in Income Breeder, CT

Significant intersite differences in the concentrations of 5 trace elements (Cr, Hg, Ni, Se, and Sr) in CT eggshells (Table 4; ES5) suggest that this species, adopting an income breeder strategy, foraged close to breeding sites in distinct areas differing in habitat composition and elemental concentrations.

In contrast to the expected elevated concentrations of fertilizer-derived Cd (Lugon-Moulin et al. 2006; Nziguheba and Smolders 2008) in the eggshells of CTs breeding in sites with a high proportion of surrounding farmland (Pakość), no significant site effect was found. This may be explained by the generally low intensity of agrochemical use in small farms prevailing in the studied area (Statistical Office in Bydgoszcz 2011). Because high Cu levels may indicate high concentrations of aquatic prey, the lack of a significant site effect for Cu levels in eggshells may be explained by the lack of significant intersite differences in the area’s water bodies (Table ES1). Significant intersite differences in the levels of five elements were found in the CT eggshells. The highest concentration of Se was found in CT eggshells at the Skoki Duże colony. However, the values found in the eggshells of all studied species were lower than those reported for other waterbirds (Table ES6, Table ES7), which was attributed to a very low Se content in the soils in Poland affecting its availability for organisms (Wasowicz et al. 2003; Nowakowska et al. 2014; Mirowski 2016). The highest levels of Se in eggshells from Skoki Duże may be explained by assimilation of this element from alternative sources, such as fish from watercourses affected by runoff from soils supplemented with Se-enriched fertilizers or by local oil/fuel spills in the vicinity of the Vistula River (Lemly 2004; Hartikainen 2005).

Significantly higher levels of Ni and Sr were found at Skoki Duże and Pakość compared with the Koronowo colony. In the case of the Skoki Duze colony, it may have been due to the foraging of CT in the Włocławek Reservoir (a water reservoir on the Vistula River) situated in close proximity (< 1 km) to the colony. The Vistula River transports multiple pollutants, including Ni (25.2 tons annually) (Polish Central Statistical Office 2013). High Sr levels at Skoki Duże is likely attributable to the colony’s location in a functioning gravel pit. Soil and parent rock are natural reservoirs of this element (Turekian and Kulp 1956; Kabata-Pendias and Mukherjee 2007) and serve as major sources of contaminated fish and other aquatic organisms. High levels of Ni (and also Cr) in the eggshells from Pakość may be attributable to emissions from a nearby factory producing machines for industry and mining often using alloys (Nriagu 1988; Studnicki et al. 2005; Duda-Chodak and Blaszczyk 2008). The highest Cr concentration was found in the colony at Pakość, which may be explained by the close proximity of a tannery. Wastewater from the tanning process is considered a major source of Cr pollution in wetland sediments as the untreated tannery effluent is characterized by high concentrations of Cr, salts, chloride ions, sulfides, and sulfates (Pawlikowski et al. 2006; Rosales et al. 2017).

### Limitations of Our Study

We are aware of some limitations of our study. First, our study is based on post-hatch eggshells. Eggshells and egg contents may have different trace-element levels (Morera et al. 1997; Agusa et al. 2005; Hashmi et al. 2013). It has been reported that Cd, Pb, and Mn concentrations in avian eggshells are higher than in egg contents (Kim and Oh 2014). Because an eggshell is mainly composed of Ca, trace elements, such as Cd and Pb, might interact with the metabolic pathway of Ca (Scheuhammer 1987). Consequently, they may be incorporated more easily in the eggshell (Dauwe et al. 1999). However, those differences may be advantageous in biomonitoring studies. Comparison of Zn and Cu concentrations in eggs of birds breeding in polluted and unpolluted areas revealed lack of differences for egg content and marked differences in eggshells. It indicates that the concentrations of both elements in the egg content are homeostatically controlled. In this context, the egg content is considered as less suitable as a bioindicator compared with the eggshell (Dauwe et al. 1999). Moreover, concentrations of some elements were reported to be similar (Cu, Mg, Mg, and Zn) or significantly correlated (Cd, Pb, Cu) in eggshell and egg content (Kim and Oh 2014). Anyway, caution should be use when interpreting results for particular elements. Second, our interpretations of the observed differences in elemental concentrations are mainly focused on dietary differences and local soil and water pollution sources. However, many other factors, such as metabolic state and health may affect the sequestration of particular elements into the egg.

Despite both mentioned limitations, analyses of contaminations levels in the post-hatched eggshells may serve as convenient, not invasive, tool for monitoring trace-element contaminations in birds (Dauwe et al. 1999; Lam et al. 2005;  Ayas 2007; Ayas et al. 2008; Kim and Oh 2014).

## Conclusions

Our study revealed significant inter-species differences in elemental concentrations in post-hatch eggshells of three sympatrically breeding waterbirds. Those differences were attributed to various diet compositions and geographic areas for gaining energy reserves for egg production. Comparisons with the eggshells of other waterbirds revealed that the studied birds generally did not accumulate high levels of toxic elements. Levels of Cr in ML and CT were exclusively elevated, which may be explained by their foraging on aquatic organisms in waterbodies polluted by this element. The results of our intersite comparisons reflecting local sources of contamination suggest that the eggshells of income breeders may be used as bioindicators of contamination levels in the vicinity of breeding sites. The decomposition of the eggshells of waterbirds serves as one of the local-scale mechanisms of pollution transfer from aquatic to terrestrial ecosystems.

## Electronic supplementary material

Below is the link to the electronic supplementary material.

Supplementary material 1 (DOCX 47 kb)
