# Supplementary material for: Factors Affecting Element Concentrations in Eggshells of Three Sympatrically Nesting Waterbirds in Northern Poland
Source: Arch Environ Contam Toxicol. 2017 Nov 23;74(2):318–29. doi: 10.1007/s00244-017-0481-y (PMC5807457; doi:10.1007/s00244-017-0481-y)
Supplement: Supplementary file 1 — Supplementary material 1 (DOCX 47 kb) [file 244_2017_481_MOESM1_ESM.docx]

**Electronic supplementary material ES1**

# Factors affecting element concentrations in eggshells of three sympatrically

**nesting waterbirds in N Poland**

Ignacy Kitowski, Dariusz Jakubas, Piotr Indykiewicz, Dariusz Wiącek

A *G* test was used to compare the proportion of the area covered by particular types of habitats in 10-km buffers around the studied sites. We extracted particular landscape features from the Corine Land Cover model CLC 2006 (European Environment Agency) using ArcMap software, version 10.3.1 (ArcGIS, ESRI, Redlands, CA). The 10-km buffer areas around the studied sites consist mainly of agricultural land (47-86%) and forest (6-42%; Table ES1).

**Table ES1** Relative contribution [%] of habitats 10 km around the studied sites [according to the Corine Land Cover (CLC2006) model (http://www.eea.europa.eu/, EEA Copenhagen, 2012)]

| Habitat type | Koronowo | Skoki Duże | Pakość | *G* test, *p* |
| --- | --- | --- | --- | --- |
| Agriculture areas | 51.6 | 47.2 | 85.8 | 0.015 |
| Urbanized area | 1.6 | 0.3 | 4.2 | 0.095 |
| Forested areas | 42.2 | 40.3 | 6.3 | <0.001 |
| Water bodies | 4.5 | 0.9 | 3.7 | 0.271 |
| Water courses | 0.1 | 11.2 | - | - |
| Wetlands | 0.02 | - | 0.08 | - |

*G* test – results of *G* test comparing the proportion of particular habitat types among the studied sites

**Details of analytical procedures**

1. Mineralisation was ran according to the following scheme: 15 min from room temperature to 140 °C, 5 min at 140 °C, 5 min from 140 °C to 170 °C, 15 min at 170 °C and cooling to room temperature (varied). Pressure did not exceed 12 bars during mineralisation.

2. To detect particular elements, the following instrumental parameters of the spectroscope were used (iCAP 6000 Series Hardware Manual 2010):

1) RF generator power 1150 W, frequency 27.12 MHz,

2) Flow rate for: coolant gas, 16 L· min−1; carrier gas, 0.65 L· min−1; auxiliary gas, 0.4 L· min−1,

3) Max integration time - 15 s,

4) Pump rate - 50 rpm,

6) Viewing configuration - axial,

7) Replicate - 3,

8) Flush time - 20 s.

Multielement stock solutions from Inorganic Ventures were used as standards:

A) Analityk - 46: Cu, Fe, Mg, P, K, Na in 5% HNO_3_ – 1,000 µg· mL^-1^,

B) Analityk - 47: Al, As, Cd, Cr, Pb, Mn, Hg, Ni, Sc, Se, Sr, V, Zn in 10% HNO_3_ - 100 µg· mL^-1^,

C) Analityk - 83: Ca, K, Mg, Na, P, S in 2% HNO3-1000 mg· L^-1^,

D) CGMO1-1: Mo in H_2_O with traces of NH_4_OH - 1000 µg· mL^-1^.

3. Validation of the analytical method.

A) Linearity (the ability of the method to obtain test results proportional to the concentration of the analyte) was calculated using Pearson correlation coefficient (Table ES2).

B) To calculate the recovery percentage, three randomly selected samples were individually supplied with known amounts of the analytical standard. The mean percentage recoveries of the analyzed elements were calculated based on the following equation: Recovery [%] = (C_E_/C_S_ * 100), where C_E_ is the experimental concentration determined from the calibration curve and C_S_ is the spiked concentration (Table ES2).

**Table ES2** Validation of the analytical method: linearity (the ability of the method to obtain test results proportional to the concentration of the analyte; *r* - Pearson correlation coefficient, detection limit, and recoveries for the studied elements

| Element | Linearity *r* | Limit of detection  LOD [µg/L] | Recovery  [%] |
| --- | --- | --- | --- |
| As | 0.9995 | 0.011 | 99 |
| Ca | 0.9985 | 0.002 | 105 |
| Cd | 0.9999 | 0.001 | 97 |
| Cr | 0.9997 | 0.003 | 97 |
| Cu | 0.9999 | 0.002 | 103 |
| Fe | 0.9998 | 0.021 | 96 |
| Hg | 0.9996 | 0.058 | 97 |
| Mg | 0.9953 | 0.005 | 104 |
| Mn | 0.9998 | 0.002 | 96 |
| Mo | 0.9996 | 0.022 | 98 |
| Ni | 0.9999 | 0.001 | 97 |
| Pb | 0.9999 | 0.010 | 98 |
| Sc | 0.9997 | 0.002 | 99 |
| Se | 0.9995 | 0.012 | 97 |
| Sr | 0.9998 | 0.003 | 98 |
| V | 0.9999 | 0.003 | 97 |
| Zn | 0.9998 | 0.010 | 102 |

**Table ES3** Results of two-way PERMANOVA analyses for particular elements (with elemental concentration as the analyzed variable and species, site and species × site interaction as predictors)

| As | *df* | *F* | *p* | Ca | df | *F* | *p* | Cd | *df* | *F* | *p* |
| --- | --- | --- | --- | --- | --- | --- | --- | --- | --- | --- | --- |
| Species | 2 | 101.7 | 0.0001 | Species | 2 | 7069.3 | 0.0001 | Species | 2 | 2.7 | 0.018 |
| Site | 2 | 1.2 | 0.242 | Site | 2 | 5.7 | 0.003 | Site | 2 | 0.9 | 0.473 |
| Interaction | 4 | -1.0 | 0.929 | Interaction | 4 | 11.4 | 0.0001 | Interaction | 4 | -0.4 | 0.421 |
| Cr | *df* | *F* | *p* | Cu | *df* | *F* | *p* | Fe | *df* | *F* | *p* |
| Species | 2 | 86.9 | 0.0001 | Species | 2 | 75.1 | 0.0001 | Species | 2 | 30.5 | 0.0001 |
| Site | 2 | 5.6 | 0.002 | Site | 2 | 1.6 | 0.157 | Site | 2 | 1.3 | 0.252 |
| Interaction | 4 | 1.5 | 0.018 | Interaction | 4 | -0.5 | 0.419 | Interaction | 4 | -0.4 | 0.399 |
| Hg | *df* | *F* | *p* | Mg | *df* | *F* | *p* | Mn | *df* | *F* | *p* |
| Species | 2 | 234.5 | 0.0001 | Species | 2 | 354.2 | 0.0001 | Species | 2 | 47.9 | 0.0001 |
| Site | 2 | 3.2 | 0.026 | Site | 2 | 2.3 | 0.095 | Site | 2 | 5.3 | 0.001 |
| Interaction | 4 | 2.9 | 0.0011 | Interaction | 4 | 3.2 | 0.0009 | Interaction | 4 | -0.1 | 0.231 |
| Mo | *df* | *F* | *p* | Ni | *df* | *F* | *p* | Pb | *df* | *F* | *p* |
| Species | 2 | 12.7 | 0.0001 | Species | 2 | 98.0 | 0.0001 | Species | 2 | 16.0 | 0.0001 |
| Site | 2 | 0.9 | 0.456 | Site | 2 | 27.5 | 0.0001 | Site | 2 | 1.7 | 0.138 |
| Interaction | 4 | -0.3 | 0.309 | Interaction | 4 | 23.0 | 0.0001 | Interaction | 4 | -0.5 | 0.438 |
| Sc | *df* | *F* | *p* | Se | *df* | *F* | *p* | Sr | *df* | *F* | *p* |
| Species | 2 | 6.3 | 0.0001 | Species | 2 | 112.7 | 0.0001 | Species | 2 | 247.9 | 0.0001 |
| Site | 2 | 3.4 | 0.004 | Site | 2 | 6.6 | 0.0008 | Site | 2 | 92.5 | 0.0001 |
| Interaction | 4 | 2.9 | 0.0001 | Interaction | 4 | 2.5 | 0.003 | Interaction | 4 | 17.9 | 0.0001 |
| V | *df* | *F* | *p* | Zn | *df* | *F* | *p* |  |  |  |  |
| Species | 2 | 39.0 | 0.0001 | Species | 2 | 69.3 | 0.0001 |  |  |  |  |
| Site | 2 | 3.0 | 0.010 | Site | 2 | 0.4 | 0.683 |  |  |  |  |
| Interaction | 4 | -0.4 | 0.406 | Interaction | 4 | 1.0 | 0.040 |  |  |  |  |

**Table ES4** Elemental concentrations (medians and min-max [mg · kg^-1^]) in the eggshells of the studied species, black-headed gull (BHG), mallard (ML), and common tern (CT)

| Element | BHG (N = 35) | |  | ML (N = 34) | |  | CT (N = 36) | | |
| --- | --- | --- | --- | --- | --- | --- | --- | --- | --- |
|  | Median | Min | Max | Median | Min | Max | Median | Min | Max |
| As | 0.42^a,b^ | ND | 0.91 | 0.05^a,c^ | 0.02 | 0.08 | 0.02^b,c^ | ND | 0.05 |
| Ca | 327.55^a,b^ | 314.26 | 340.57 | 127.24^a,c^ | 111.27 | 142.10 | 134.20^b,c^ | 113.50 | 145.33 |
| **Cd** | 0.01 | ND | 0.05 | 0.02^a^ | ND | 0.03 | 0.01^a^ | ND | 0.09 |
| **Cr** | 1.55^a,b^ | 0.51 | 4.84 | 4.46^a,c^ | 2.55 | 15.72 | 5.86^b,c^ | 4.59 | 9.72 |
| Cu | 0.66^a,b^ | 0.03 | 1.59 | 4.89^a,c^ | 1.56 | 10.48 | 3.21^b,c^ | 1.68 | 9.09 |
| Fe | 17.76^a,b^ | 8.67 | 109.77 | 2.53^a,c^ | 0.32 | 31.26 | 9.68^b,c^ | 0.17 | 262.7 |
| **Hg** | 0.00^a,b^ | ND | 0.00 | 0.21^a,c^ | 0.15 | 0.27 | 0.14^b,c^ | ND | 0.52 |
| Mg | 2,025^a^ | 1,745 | 2,993 | 1,093^a,b^ | 880 | 14,001 | 2,044^b^ | 1,546 | 2,655 |
| Mn | 0.94^a,b^ | 0.48 | 3.2 | 3.61^a,c^ | 1.17 | 10.92 | 0.58^b,c^ | 0.08 | 6.31 |
| **Mo** | 0.08^a,b^ | ND | 0.2 | 0.03^a,c^ | 0.01 | 0.08 | 0.03^b,c^ | ND | 0.31 |
| **Ni** | 0.26^a,b^ | 0.19 | 0.32 | 0.44^a,c^ | 0.15 | 2.43 | 0.16^b,c^ | 0.04 | 0.25 |
| **Pb** | 0.45^a^ | 0.20 | 1.72 | 0.89^a,b^ | 0.47 | 1.61 | 0.52^b^ | 0.11 | 1.15 |
| **Sc** | 0.03^a^ | ND | 0.05 | 0.02^b^ | 0.01 | 39.68 | 0.04^a,b^ | 0.02 | 0.24 |
| Se | 1.68^a,b^ | 1.07 | 2.55 | 0.74^a,c^ | 0.21 | 1.07 | 1.39^b,c^ | 0.89 | 3.37 |
| Sr | 245.63^a^ | 151.49 | 367.64 | 255.00^b^ | 122.2 | 485.45 | 124.17^a,b^ | 45.97 | 176.23 |
| **V** | 0.07^a,b^ | ND | 0.20 | 1.01^a,c^ | 0.26 | 2.11 | 0.03^b,c^ | ND | 0.49 |
| Zn | 32.04^a,b^ | 9.36 | 53.42 | 6.17^a,c^ | 1.93 | 28.77 | 12.54^b,c^ | 6.16 | 40.18 |

^a,b,c^Significant interspecies differences, PERMANOVA on log(x+1) transformed data, *p* < 0.05

*ND* not detected

Heavy metals bolded **Table ES5** Elemental concentrations (medians and min-max [mg ⋅ kg^-1^]) in the eggshells of the common terns from the studied sites (only elements with significant intersite differences presented)

| Element | Skoki Duże (N = 12) | | | Koronowo (N = 12) | | | Pakość (N = 12) | | |
| --- | --- | --- | --- | --- | --- | --- | --- | --- | --- |
|  | Median | Min | Max | Median | Min | Max | Median | Min | Max |
| Cr | 5.78 | 4.95 | 6.63 | 5.50^a^ | 4.59 | 6.27 | 6.70^a^ | 5.11 | 9.72 |
| Hg | 0.17 | 0.00 | 0.25 | 0.05^a^ | 0.00 | 0.52 | 0.16^a^ | 0.08 | 0.25 |
| Ni | 0.21^a^ | 0.13 | 0.24 | 0.07^a,b^ | 0.04 | 0.16 | 0.20^b^ | 0.15 | 0.25 |
| Se | 1.85^a,b^ | 1.55 | 3.37 | 1.26^a^ | 0.89 | 1.43 | 1.17^b^ | 0.92 | 1.70 |
| Sr | 139.10^a^ | 121.13 | 169.56 | 61.99^a,b^ | 45.97 | 104.40 | 138.22^b^ | 109.36 | 176.23 |

^a,b^Significant intersite differences

PERMANOVA on log(x+1) transformed data, *p* < 0.05 **Table ES6** Cadmium, chromium, nickel, lead, copper, and zinc concentrations (mg ⋅ kg^-1^ dw) in the eggshells of waterbirds from various studies (see references and species codes in Table ES7)

| Species | Cd | Species | Cr | Species | Ni | Species | Pb | Species | Cu | Species | Mn | Species | Zn |
| --- | --- | --- | --- | --- | --- | --- | --- | --- | --- | --- | --- | --- | --- |
| AG | ND | BCH2 | 0.32 | CP | 0.03 | AG | ND | BTG2 | 0.54 | AG | 0.29 | BTG2 | 0.78 |
| LE2 | 0.001 | LE2 | 0.38 | BT2 | 0.05 | BCH2 | 0.007 | **BHG** | 0.66 | **CT** | 0.58 | AO | 2.08 |
| BT1 | 0.002 | BTG2 | 0.40 | LCT | 0.05 | BT1 | 0.010 | BHG3 | 1.37 | **BHG** | 0.94 | **ML** | 6.17 |
| LCT | 0.004 | AO | 0.78 | **CT** | 0.16 | LE2 | 0.01 | BCH3 | 1.69 | BTG2 | 1.13 | AG | 6.58 |
| CP | 0.004 | **BHG** | 1.55 | BHG3 | 0.22 | BTG2 | 0.06 | AO | 1.97 | LE2 | 1.49 | **CT** | 12.54 |
| BT2 | 0.005 | BT1 | 1.95 | BCH3 | 0.223 | LCT | 0.08 | AG | 2.14 | BCH2 | 1.72 | RCC | 18.56 |
| **BHG** | 0.010 | GE | 3.71 | **BHG** | 0.26 | BT2 | 0.24 | BTG1 | 2.80 | BT1 | 2.64 | BHG2 | 24.21 |
| **CT** | 0.010 | SG | 3.84 | GH | 0.41 | CP | 0.25 | BCH1 | 3.01 | BTG1 | 2.76 | BHG1 | 29.71 |
| BTG2 | 0.013 | BCH1 | 4.13 | **ML** | 0.44 | **BHG** | 0.45 | RCC | 3.10 | **ML** | 3.61 | **BHG** | 32.04 |
| **ML** | 0.020 | LE1 | 4.46 | AO | 6.06 | **CT** | 0.52 | **CT** | 3.21 |  |  | BCH2 | 39.87 |
| BHG3 | 0.230 | **ML** | 4.46 | SG | 19.38 | **ML** | 0.89 | GE | 3.22 |  |  | BT1 | 47.62 |
| BCH3 | 0.230 | **CT** | 5.86 | BCH1 | 23.19 | RCC | 0.92 | BT1 | 3.92 |  |  | LE2 | 51.38 |
| RCC | 0.330 |  |  | GE | 24.16 | BCH3 | 1.108 | SG | 3.93 |  |  | BTG1 | 53.60 |
| BTG1 | 0.450 |  |  | LE1 | 24.71 | BHG3 | 1.11 | LE1 | 4.05 |  |  | SG | 55.82 |
| GH | 0.931 |  |  |  |  | BTG1 | 3.10 | **ML** | 4.89 |  |  | GE | 60.08 |
| BHG1 | 5.210 |  |  |  |  | GH | 4.57 | BCH2 | 6.29 |  |  | BCH1 | 65.58 |
| BHG2 | 5.250 |  |  |  |  | AO | 8.12 | LE2 | 6.58 |  |  | LE1 | 66.51 |
| SG | 7.720 |  |  |  |  | BHG2 | 66.21 | GH | 6.76 |  |  |  |  |
| LE1 | 7.780 |  |  |  |  | BHG1 | 66.41 | BHG2 | 7.98 |  |  |  |  |
| GE | 8.080 |  |  |  |  | SG | 78.72 | BHG1 | 9.64 |  |  |  |  |
| BCH1 | 9.120 |  |  |  |  | GE | 82.14 |  |  |  |  |  |  |
| AO | 11.620 |  |  |  |  | BCH1 | 84.85 |  |  |  |  |  |  |
|  |  |  |  |  |  | LE1 | 88.51 |  |  |  |  |  |  |
|  |  |  |  |  |  |  |  |  |  |  |  |  |  |
|  |  |  |  |  |  |  |  |  |  |  |  |  |  |
|  |  |  |  |  |  |  |  |  |  |  |  |  |  |

Results from this study bolded

*ND* not detected **Table ES7** Sources of data about elemental concentrations and species codes for Table ES6

| Species code | Species | Habitat and study area | Reference |
| --- | --- | --- | --- |
| GH* | Grey heron *Ardea cinerea* | Dam reservoir, C Turkey | Ayas 2007^1^ |
| LE1* | Little egret *Egretta garzetta* | Wetlands, E China | Fu et al. 2014^2^ |
| GE* | Great egret *Ardea alba* | Wetlands, E China | Fu et al. 2014^2^ |
| BCH1* | Black-crowned night heron *Nycticorax nycticorax* | Wetlands, E China | Fu et al. 2014^2^ |
| BCH2** | Black-crowned night heron *Nycticorax nycticorax* | Coastal areas, Hong Kong | Lam et al. 2005^2^ |
| BCH3* | Black-crowned night heron *Nycticorax nycticorax* | Dam reservoir, C Turkey | Ayas 2007^1^ |
| SG* | Saunders's gull *Larus saundersi* | Wetlands, E China | Fu et al. 2014^2^ |
| BTG1** | Black-tailed gull *Larus crassirostris* | Island in the Yellow Sea, S Korea | Kim and Oh 2014^2^ |
| BTG2 | Black-tailed gull *Larus crassirostris* | Island in the Sea of Japan, N Japan | Agusa et al. 2005^2^ |
| LE2** | Little egret *Egretta garzetta* | Coastal areas, Hong Kong | Lam et al. 2005^2^ |
| BT1** | Bridled tern *Onychoprion anaethetus* | Coastal areas, Hong Kong | Lam et al. 2005^2^ |
| BT2** | Bridled tern *Onychoprion anaethetus* | Bay in Persian Gulf, S Iran | Khademi et al. 2015^3^ |
| AO** | American oystercatcher *Haematopus palliatus* | Coastal plain estuary, E Argentina | Simonetti et al. 2015^3^ |
| LCT** | Lesser crested tern *Thalasseus bengalensis* | Bay in Persian Gulf, S Iran | Khademi et al. 2015^3^ |
| CP** | Crab plover *Dromas ardeola* | Bay in Persian Gulf, S Iran | Khademi et al. 2015^3^ |
| RCC* | Red-crowned crane *Grus japonensis* | Large marshlands, NE China | Luo et al. 2016^1^ |
| AG** | Audouin's gull *Ichthyaetus audouinii* | Ebro river delta, N Spain | Morera et al. 1997^1^ |
| BHG1** | Black-headed Gull *Chroicocephalus ridibundus* | Industrial highly polluted area, S Poland | Migula et al. 2000^1^ |
| BHG2** | Black-headed Gull *Chroicocephalus ridibundus* | Industrial less polluted area, S Poland | Migula et al. 2000^1^ |
| BHG* | Black-headed Gull *Chroicocephalus ridibundus* | Small lakes and reservoirs, N Poland | This study^3^ |
| CT* | Common tern *Sterna hirundo* | Small lakes and reservoirs, N Poland | This study^3^ |
| ML* | Mallard *Anas platyrhynchos* | Small lakes and reservoirs, N Poland | This study^3^ |

^1^Geometric mean

^2^Aritmetic mean

^3^Median

*Shells of embryonated eggs; **shells of nonembryonated eggs
